# A smartphone-enabled wireless and batteryless implantable blood flow sensor for remote monitoring of prosthetic heart valve function

**DOI:** 10.1371/journal.pone.0227372

**Published:** 2020-01-14

**Authors:** Bernhard Vennemann, Dominik Obrist, Thomas Rösgen

**Affiliations:** 1 Institute of Fluid Dynamics, ETH Zürich, Zürich, Switzerland; 2 ARTORG Center for Biomedical Engineering Research, University of Bern, Bern, Switzerland; Case Western Reserve University Jack Joseph and Morton Mandel School of Applied Social Sciences, UNITED STATES

## Abstract

Aortic valve disease is one of the leading forms of complications in the cardiovascular system. The failing native aortic valve is routinely surgically replaced with a bioprosthesis. However, insufficient durability of bioprosthetic heart valves often requires reintervention. Valve degradation can be assessed by an analysis of the blood flow characteristics downstream of the valve. This is cost and labor intensive using clinical methodologies and is performed infrequently. The integration of consumer smartphones and implantable blood flow sensors into the data acquisition chain facilitates remote management of patients that is not limited by access to clinical facilities. This article describes the characteristics of an implantable magnetic blood flow sensor which was optimized for small size and low power consumption to allow for batteryless operation. The data is wirelessly transmitted to the patient’s smartphone for in-depth processing. Tests using three different experimental setups confirmed that wireless and batteryless blood flow recording using a magnetic flow meter technique is feasible and that the sensor system is capable of monitoring the characteristic flow downstream of the valve.

## Introduction

Aortic valve (AV) disease is a common pathology of the cardiovascular system and often appears in form of structural valve deterioration, endocarditis, pannus growth, or valve thrombosis [1]. These pathologies can lead to a narrowing of the valve’s orifice area (aortic stenosis), or to leaking of the valve due to incomplete coaptation of the leaflets during diastole (aortic regurgitation) [2]. For moderate to severe cases of aortic stenosis or aortic regurgitation, the diseased AV is often replaced by a bioprosthetic heart valve (BHV) which aims at restoring physiological blood flow [3]. BHVs, however, suffer from reduced durability such that reinterventions may be required. The rate at which BHVs degrade over time varies strongly among patients and renders prediction of valve durability difficult [4]. Current clinical diagnostic modalities often rely on the measurement of blood flow because it serves as an important indicator of cardiovascular health. These medical examinations, however, are labor and cost intensive and are performed infrequently. Medical sensory implants used in conjunction with the patient’s smartphone can allow for remote monitoring of BHV function at frequent intervals with minimal overhead through a telemedicine approach and can be used to automate the data acquisition process. Such blood flow measurements taken immediately downstream of the AV can yield important information for the early diagnosis of valvular defects and BHV degradation. In recent years, several systems for implantable large-vessel blood flow meters based on Doppler ultrasound technology have been published [5–11]. Other studies have been published where blood flow is determined from blood pressure or the dilation of the blood vessel [12–16]. The development of magnetic blood flow meters has been mostly limited to wired devices for intraoperative flow monitoring [17]. One reason can be seen in the high power demands of the magnetic field producing solenoids, and such high power requirements of many flow measurement techniques have been suggested as the main limitation of current implantable blood flow sensing devices [18]. An optimized arrangement of multiple permanent magnets [19, 20] allows redesigning the classical electromagnetic flow meter for reduced power consumption. This includes replacement of the power-demanding electromagnetic field coils with a set of permanent magnets (which require no energy) to produce a directed magnetic field and the design of an electronic circuit using low-power components. Here, we propose an implantable blood flow sensing technique that is based on the principles of a magnetic blood flow sensor which was modified using permanent magnets to enable low-power operation suitable for medical implants. This crucial modification enables batteryless and wireless operation at an implantable scale for long-term application in the human body. The article describes the functioning and the characteristics of this wireless and batteryless implantable blood flow sensor that was designed for remote monitoring of bioprosthetic heart valve function.

## Materials and methods

### Device description

**Fig 1:**
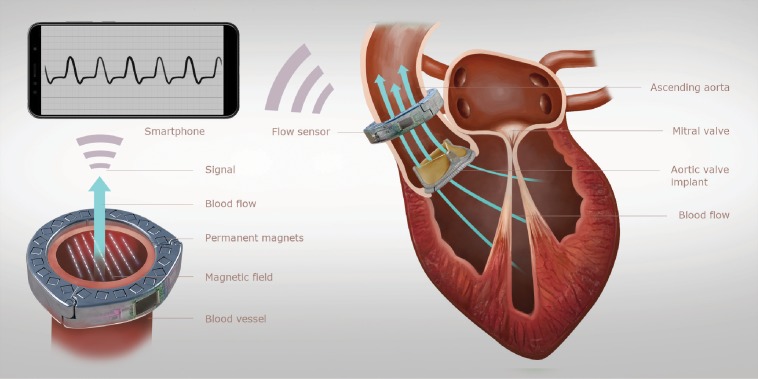
Illustration of the heart valve monitoring system. An implantable magnetic flow sensor attaches to the ascending aorta to measure the characteristic flow profile downstream of the aortic valve. A smartphone wirelessly receives the measurement data and inductively powers the implant.

**Fig 2:**
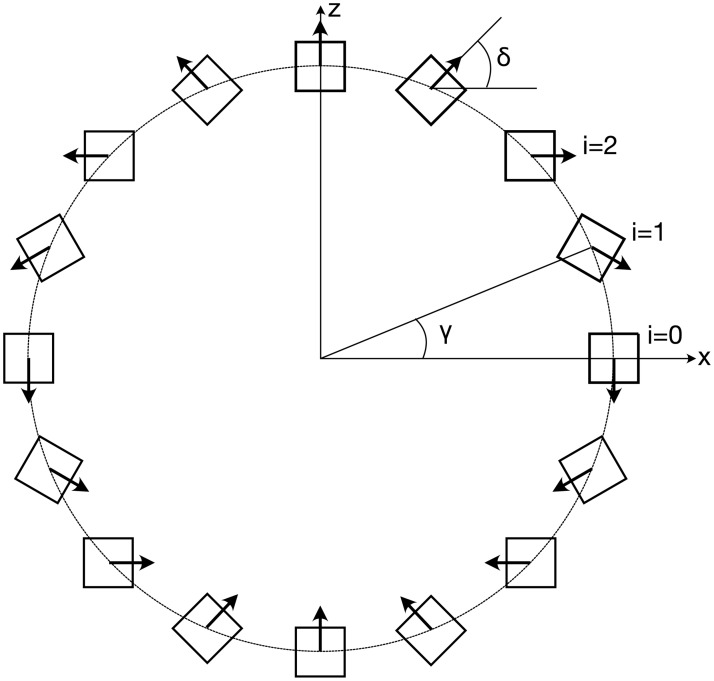
Design principle of a circular Halbach array. The angles *γ* and *δ* can be computed according to Eqs 2 and 3.

Fig 1 shows an illustration of the working principle of the sensor system that was developed for prosthetic heart valve monitoring. The measurement system comprises two components. The first component is an implantable blood flow sensor in the shape of a perivascular cuff which can be attached to the ascending aorta. The second component is a consumer smartphone that is used as extracorporeal energy source and provides the wireless data link between the implant and the outside world. Semi-continuous recordings of the characteristic patterns of blood flow rate downstream of the AV using this system can help early detection of AV degradation through an automated long-term analysis of this data. The flow sensing implant utilizes the principles of a magnetic flow sensor, a technology which has been successfully applied for the measurement of blood flow as early as the 1930s [21, 22]. It exploits Faraday’s law of induction which states that a conductor moving through a directed magnetic field will experience an electromotive force (EMF) that is proportional to the velocity of the conductor and the strength of the magnetic field. In the context of blood flow measurement, this conductor is the human blood whose velocity may be determined by applying an external directed magnetic field and by measuring the voltage differential between electrodes that are in contact with the vessel wall on opposing sides of the blood vessel and perpendicular to the direction of the magnetic field. This signal is directly proportional to the average velocity of blood flow within the vessel and can be used in conjunction with the known vessel diameter to determine the instantaneous flow rate [23]. This relationship is often expressed using a simplified flow meter equation

$$E=B\bar{u}d,$$ (1)

where *E* [*V*] is the induced EMF, *B* [*T*] is the uniform magnetic flux density, $\bar{u}\;\left[m/s\right]$ is the average velocity in the blood vessel and *d* [*m*] is the electrode distance (vessel diameter) [17]. The directed magnetic field is typically generated with a set of electromagnetic field coils, and this technique has been also used for blood flow sensors. However, the high energy demand of these solenoids prevents wireless and batteryless applications [24]. Our sensor system uses an approach that replaces the solenoids with an arrangement of permanent magnets to drastically reduce the energy demands of the device and to enable fully wireless and batteryless operation. Halbach [20] has shown that a directed magnetic field similar to that created by the field coils can be generated using a circular array of permanent magnets. These configurations produce a uniform magnetic field inside the array and a vanishing field outside, such that the field is contained within the magnet ring. Halbach proposed the use of circular segment magnets for this configuration, but similar properties can also be achieved using simple bar magnets [25]. The magnets are rotated along the longitudinal axis such that their respective polarization vector performs a 180 degree rotation per quadrant (Fig 2), where the magnet count *N* must be a multiple of four to obtain a symmetrical array. The circumferential position *γ* and the orientation *δ* of each magnet in the array can be computed as

$$\gamma_{i}=\frac{\pi }{2}\frac{4}{N}i$$ (2)

$$\delta_{i}=\pi \frac{4}{N}i-\frac{\pi }{2},$$ (3)

where *i* = 0, …, *N* − 1 denotes the *i*^th^ magnet in the array (cf. Fig 2).

**Fig 3:**
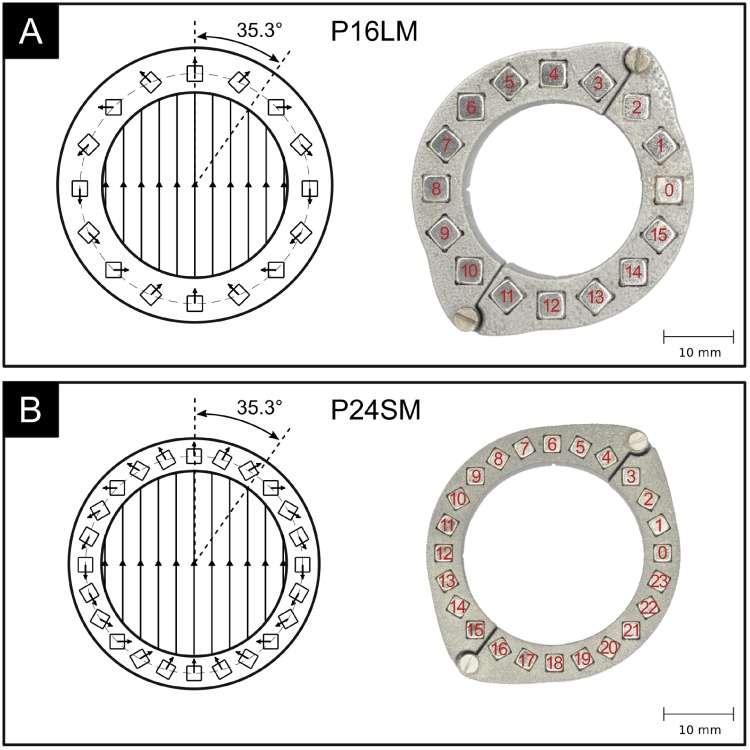
Two prototype Halbach ring implementations. A: using 16 neodymium magnets of size 4mm x 4mm x 15mm. B: using 24 neodymium magnets of size 2.5mm x 2.5mm x 7.5mm. Inner diameter: 26mm.

Fig 3 shows two prototype magnet arrays in a Halbach ring configuration. The first prototype (Fig 3A) comprises a 3D-printed stainless steel ring that hosts 16 neodymium magnets of size 4mm x 4mm x 15mm and a remanent magnetization of *B*_*r*_ ≈ 1.35 T. The second prototype (Fig 3B) was optimized for smaller size and weight and uses a 3D-printed titanium ring that hosts 24 neodymium magnets of size 2.5mm x 2.5mm x 7.5mm and *B*_*r*_ ≈ 1.35 T. In the following, we will refer to these two prototype magnet rings as: “**P**rototype **16** **L**arge **M**agnets (P16LM)” and “**P**rototype **24** **S**mall **M**agnets (P24SM)”, respectively. The size and the number of magnets should be chosen such as to maximally utilize the available space, and thereby maximizing the Halbach ring fill factor which can be computed for magnets of quadratic cross-section as

$$\rho =\frac{Nw^{2}}{\pi (R_{o}^{2}-R_{i}^{2})},$$ (4)

where *w* is the magnet side length, and *R*_*i*_ and *R*_*o*_ are the the ring’s inner and outer radius. This maximizes the amount of magnetic material for a given ring size and thereby the magnetic flux density within the lumen. A high magnetic flux density is desirable because the flow signal magnitude scales proportionally (see Eq 1). There exists a trade-off between the strength of the magnetic field and the size of the resulting Halbach ring, where smaller rings yield weaker magnetic fields. The magnet ring should be designed to produce a sufficiently strong magnetic field while maintaining a footprint suitable for implantation. The normal force between adjacent magnets is minimal at an angle of 35.3 degrees (and 215.3 degrees) with respect to the direction of the magnetic field. This angle had been derived from a two-dimensional, continuous distribution of magnetic moments in the array and considering only the direct influence of adjacent dipoles [26]. It can be regarded as a reasonable approximation of the optimal angle for arrays constructed from discrete bar magnets. The ring can be opened with minimal force when the opening mechanism is located at these angles. The opening location between two magnets that approaches the optimum opening angle of 35.3 degrees is between magnets 2 and 3 for the 16-magnet array and between magnets 3 and 4 for the 24-magnet array (cf. Figs 2 and 3).

**Fig 4:**
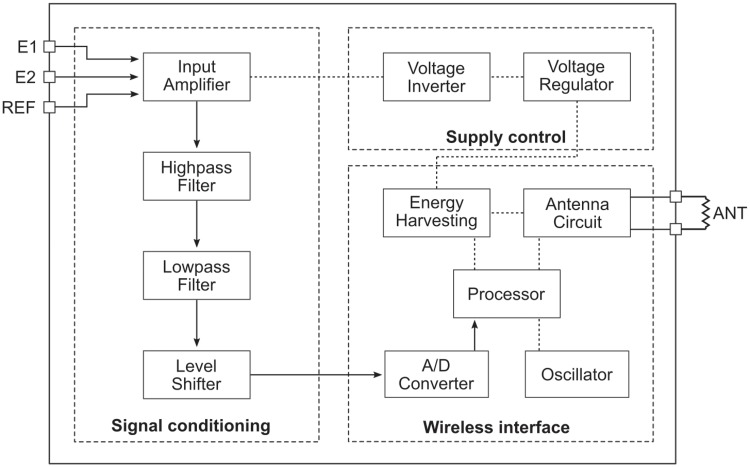
Functional block diagram of the electronic circuit. The circuit comprises three main functional blocks: signal conditioning, wireless interface and supply control. The path of the blood flow signal is indicated by arrows.

As blood is a conductive liquid, it will experience an EMF when it passes through the magnet array. This produces a voltage differential across the fluid which can be picked up with electrodes in contact with the outside of the conductive vessel wall on opposing sides of the blood vessel and orthogonal to the direction of the magnetic field. This signal is on the order of 1 mV for typical cardiac flow rates and the prototype arrays shown in Fig 3. The raw signal is amplified and filtered before it is fed into the analog-to-digital converter (ADC) and subsequently wirelessly transmitted to the patient’s smartphone. The device has been designed for bidirectional flow measurement to record forward and reverse flow through the aorta (and the AV). The functional block diagram of the electronic circuitry is depicted in Fig 4. The circuit consists of three main functional blocks: signal conditioning, supply control, and a wireless interface. The raw signal is connected to the circuit through the positive and negative leads E1, E2 and a reference electrode REF (perpendicular to E1 and E2 in the magnet array). The raw signal is processed in the signal conditioning block. Here, the signal is amplified and filtered to remove undesirable signal components. An input instrumentation amplifier (INA321 Micropower single-supply CMOS Instrumentation Amplifier, Texas Instruments Inc., Dallas, USA) provides an initial amplification of the raw signal before it is fed to a bandpass filter that consists of a highpass filter, followed by a lowpass filter stage and uses two low-power operational amplifiers (OPA336 Mircopower single-supply CMOS Operational Amplifier, Texas Instruments Inc., Dallas, USA). The bandpass filter removes any DC-offset or drift from the signal, attenuates high-frequency noise above 30 Hz, and provides additional amplification. The signal is finally processed in a level shifter using another OPA336 to match the input range of the ADC. A wireless interface block enables the digital communication between the implant and the smartphone and provides the capability for energy harvesting from the RF-field. It is based on a near field communication (NFC) chip (SL13A Smart Sensory Tag, ams AG, Premstaetten, Austria) such that it can work with any modern smartphone (with built-in NFC functionality) without additional hardware. The NFC chip features the possibility to harvest energy from the smartphone’s RF-field. This wireless power transfer is based on inductive coupling between the smartphone’s built-in NFC antenna and the antenna of the implant. The smartphone acts as the primary device and creates an oscillating electromagnetic field through its NFC antenna. When a secondary coil (implant antenna) is brought into vicinity of the primary coil, a voltage is induced which is rectified to drive an electronic DC-load. The implant’s NFC chip creates a rectified voltage of 3.4 V and can provide up to 4 mA of current (depending of the RF-field source) to drive the flow-sensing circuitry. An external sensor interface of the NFC chip enables wireless transmission of blood flow recordings.

**Fig 5:**
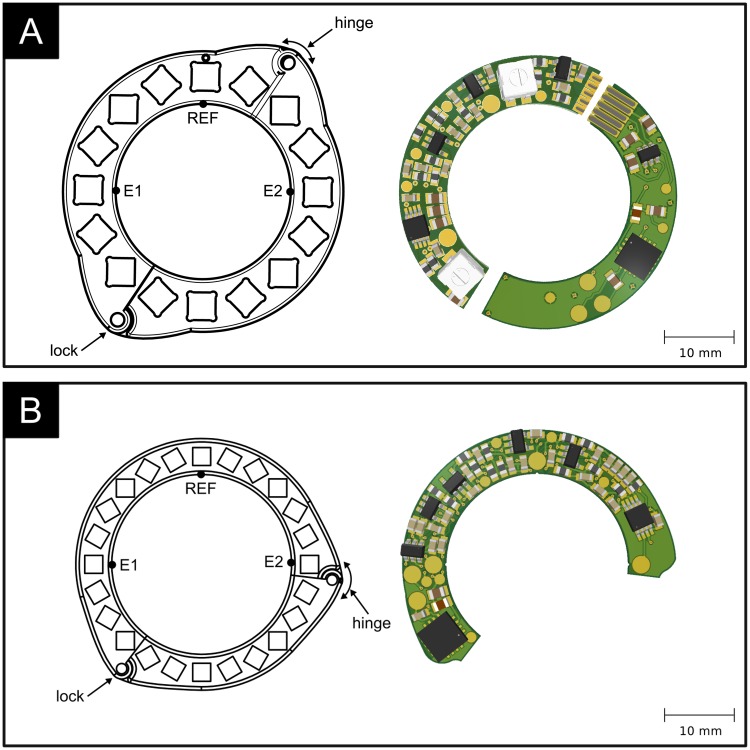
Two implementations of the electronic circuitry on a printed circuit board (PCB). A: using two semicircular PCBs that are fitted onto each half of the magnet array and connected through gold-plated electrical spring contacts. B: using a single PCB and an alternatively segmented magnet ring.

The harvested energy is conditioned in a supply control block where a rectified supply voltage of 3.4 V and its corresponding inverse voltage for bipolar operation are created. These are used to power all active components of the electronic circuit to enable batteryless operation. Fig 5 shows two implementations of the circuitry on a printed circuit board (PCB). It can take shape of two semicircular PCBs that are connected through gold-plated spring contacts (Fig 5A), but the circuitry and the ring’s sectioning can also be modified to employ only a single PCB which greatly reduces the electronic layout complexity (Fig 5B). The magnet arrangement of the latter design features a c-shaped section that holds the PCB and a smaller closing piece at the optimal opening angle to complete the Halbach ring. The design using two semicircular PCBs was developed for simplified laboratory testing as it can be easily attached to rigid tubing, whereas the second design requires slight pinching of the compliant vessel for sensor attachment and was developed for in-vivo usage.

An Android^™^ smartphone application was developed for wireless communication with the implant. The application sends requests to the implant’s NFC chip using the ISO 15693 standard to retrieve measurement samples from the flow meter. This protocol yields one sample, encoded in a response message, for each transmitted request message. The effective sample rate is therefore determined by the speed at which the data frames can be encoded, transmitted and decoded. At the same time, the implant is wirelessly powered through inductive coupling between the smartphone’s built-in NFC antenna and the antenna that is included in the implant. Wireless communication and power transfer were tested using a consumer smartphone (Google Pixel XL, Alphabet Inc., Mountain View, CA, USA) and a standard thin, flexible NFC antenna of size 34 mm x 46 mm. Wireless and batteryless operation could be sustained at a distance between smartphone and antenna up to 3 cm, enabling subdermal or subcutaneous antenna placement. Placing a hand in between the smartphone and the device antenna to simulate human tissue interference showed no significant effect. The current consumption of the device is 380 μA (150 μA for NFC chip and 230 μA for flow sening circuitry) at a rectified supply voltage of 3.4 V, resulting in a device power consumption of 1.3 mW.

The smartphone data can be further transmitted to a computer system for in-depth data processing and automated detection of adverse valvular behavior using machine learning. The functioning of the computer monitoring system is described in greater detail in [27, 28].

### Magnetic field measurements

**Fig 6:**
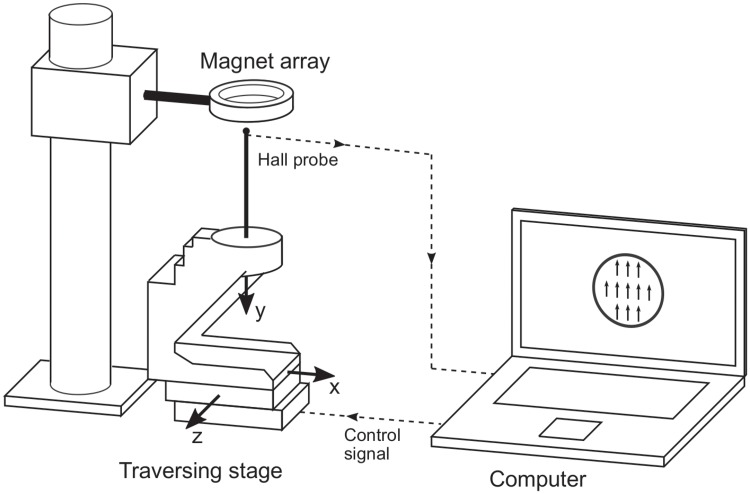
Experimental setup for magnetic field measurements. A 3-axis Hall sensor is mounted onto a computer-controlled traversing stage allowing to obtain measurements on a finely resolved grid within the magnet ring.

A test setup as illustrated in Fig 6 was used to assess the magnetic field properties of the prototype arrays. A 3-axis Hall effect sensor (THM 1176-HF, Metrolab Technology SA, Geneva, Switzerland) was mounted onto a computer-controlled precision traversing stage (SLS series, SmarAct GmbH, Oldenburg, Germany) which allowed taking magnetic field measurements inside the magnet array on a finely resolved grid. The measurements were limited to the region within the magnet array that contributes to the flow signal which corresponds to the lumen of the blood vessel and covers a circular region of 20 mm diameter in the center of the ring. Within this region, 305 measurements with a grid spacing of Δ*x* = Δ*z* = 0.91 mm were recorded in the center plane (at *y* = 0) of the magnet ring. The measurement results were compared to simulated magnetic field components *B*_*x*_ and *B*_*z*_ which were computed by superposition of the fundamental solutions for each bar magnet in the array as given in [29]

$$B_{x}\left(x,y,z\right)=\frac{\mu_{0}B_{r}}{4\pi }\sum_{k=1}^{2}\sum_{m=1}^{2}{\left(-1\right)}^{k+m}\text{ln}[F(x,y,z,x_{m},y_{1},y_{2},z_{k})],$$ (5)

where

$$F=\frac{\left(y-y_{1}\right)+{\left[{\left(x-x_{m}\right)}^{2}+{\left(y-y_{1}\right)}^{2}+{\left(z-z_{k}\right)}^{2}\right]}^{1/2}}{\left(y-y_{2}\right)+{\left[{\left(x-x_{m}\right)}^{2}+{\left(y-y_{2}\right)}^{2}+{\left(z-z_{k}\right)}^{2}\right]}^{1/2}}$$ (6)

and

$$\begin{array}{cc} B_{z}\left(x,y,z\right) & =\frac{\mu_{0}B_{r}}{4\pi }\sum_{k=1}^{2}\sum_{n=1}^{2}\sum_{m=1}^{2}{\left(-1\right)}^{k+n+m}\\ & \times {\text{tan}}^{-1}[\frac{\left(x-x_{n}\right)\left(y-y_{m}\right)}{\left(z-z_{k}\right)}g\left(x,y,z,x_{n},y_{m},z_{k}\right)], \end{array}$$ (7)

where

$$g=\frac{1}{{\left[{\left(x-x_{n}\right)}^{2}+{\left(y-y_{m}\right)}^{2}+{\left(z-z_{k}\right)}^{2}\right]}^{1/2}}.$$ (8)

Here, *μ*_0_ is the free space permeability, *B*_*r*_ is the remanent magnetization, and *x*_1_, *x*_2_, *y*_1_, *y*_2_, *z*_1_, *z*_2_ are the magnet’s corner coordinates. Furlani [29] described the derivation of these expressions based on a charge model, in which a magnet is reduced to a distribution of equivalent charge which is used as source term in the magnetostatic field equations for current-free regions

∇×H=0 (9)

∇·B=0, (10)

where **H** is the magnetic field intensity and **B** is the magnetic flux density. From this, a general solution for the magnetic flux density field is derived

$$B\left(x\right)=\frac{\mu_{0}}{4\pi }\int_{V}\frac{\rho_{m}\left(x^{\prime }\right)\left(x-x^{\prime }\right)}{|x-x^{\prime }{|}^{3}}dv^{\prime }+\frac{\mu_{0}}{4\pi }\oint_{S}\frac{\sigma_{m}\left(x^{\prime }\right)\left(x-x^{\prime }\right)}{|x-x^{\prime }{|}^{3}}ds^{\prime },$$ (11)

where *ρ*_*m*_ = −∇ ⋅ **M** is the volume charge density with magnetization **M**, $\sigma_{m}=M\cdot \hat{n}$ is the surface charge density on the surface with unit normal $\hat{n}$ and **x**′ is a source point. Integration of the general solution for a single bar magnet, centered at the origin finally yields the magnetic flux density components given in Eqs 5 and 7. The solution for the complete Halbach array is found by superposition of the contributions of each magnet within the array.

### Electronic circuit characterization

**Fig 7:**
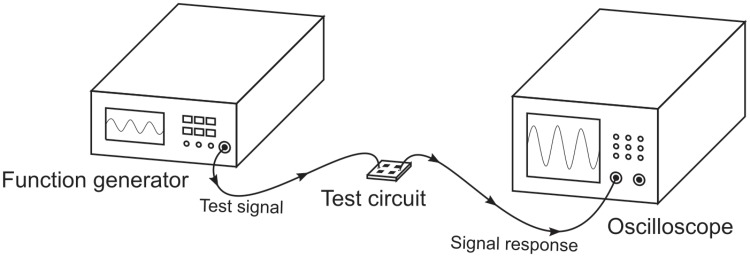
Experimental setup for electronic circuit characterization. A function generator generates sinusoidal test signals that are fed to the test circuit. Its signal response is recorded using an oscilloscope.

The frequency-dependent gain of the amplification and filtering circuitry was tested with the configuration depicted in Fig 7. Sinusoidal test signals of constant amplitude of 50mV_pp_ and variable frequencies were generated using a function generator (HP33120A, Hewlett-Packard Company, Loveland, USA), and this signal was fed to the input terminals of the test circuit (terminals E1 and E2; REF tied to ground). The output signal was recorded with an oscilloscope (Agilent 54624A, Keysight technologies, Santa Rosa, USA) to determine the amplitude of the signal response for each test frequency. A frequency sweep over a range of 0.05 Hz to 200 Hz yielded the test circuit’s transfer function for the target range.

### Flow benchmark measurements

**Fig 8:**
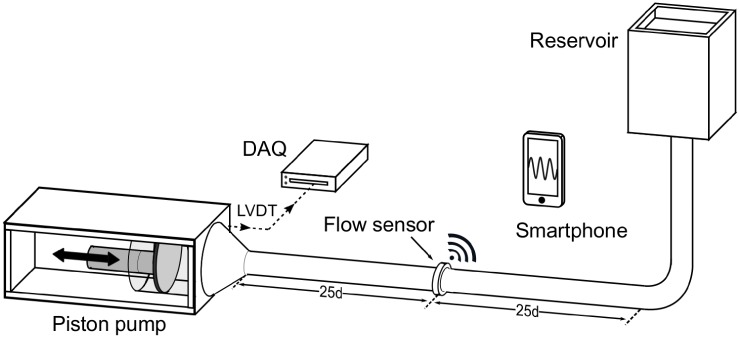
Experimental setup for end-to-end flow sensor testing. Sinusoidal flow is created using a piston pump. Reference measurements of the piston movement using an LVDT are compared to the smartphone flow recordings to determine the sensor characteristics.

The characteristics of the full measurement system, including signal generation, conditioning and wireless transmission were tested in a setup that allowed to produce well-defined flows using a computer-driven piston pump (Fig 8). The piston pump produced sinusoidal flow profiles of various amplitudes at a physiological frequency of 1 Hz (60 BPM). The flow sensor was attached to a segment of silicone tubing, connected to the piston pump with 25 diameters of straight tubing upstream and downstream of the sensor to ensure undisturbed flow. Concentric needle electrodes penetrating the non-conductive tubing were used for galvanic contact between the flow sensor and the test fluid. The fluid was an aqueous saline solution that matched the electrical conductivity of human blood of approximately 5 mS/cm [30]. The piston movement was recorded using a linear variable differential transformer (LVDT), and it was used to compute the instantaneous reference flow rate (piston velocity multiplied with cross-sectional area). The flow was simultaneously recorded with the blood flow sensing system and was saved to the smartphone’s internal memory. For prototype testing, a small flexible 13.56 MHz antenna was connected to the sensor’s PCB to provide the wireless data link. The flow sensing implant was operated in a fully self-sustained, batteryless and wireless mode through the inductive link between the implant and the smartphone. The measurements were performed at peak flow rates between -24.5 L/min and 24.5 L/min to cover a wide range of typical flow rates in the ascending aorta (a change in sign corresponds to a change in flow direction). For each amplitude setting, 10 recordings were obtained with the smartphone, and the reference LVDT signal was recorded using a data acquisition module (NI USB-6211, National Instruments Corporation, Austin, USA). The amplitudes of the sinusoidal signals recorded by the wireless flow sensor and the reference LVDT were extracted using an in-house computer code. These measurements were performed for both prototype magnet rings shown in Fig 3. A comparison of the smartphone recording to the reference signal at different amplitudes yielded the sensor characteristics: repeatability, sensitivity, linearity and resolution.

## Results

### Magnetic field properties

**Fig 9:**
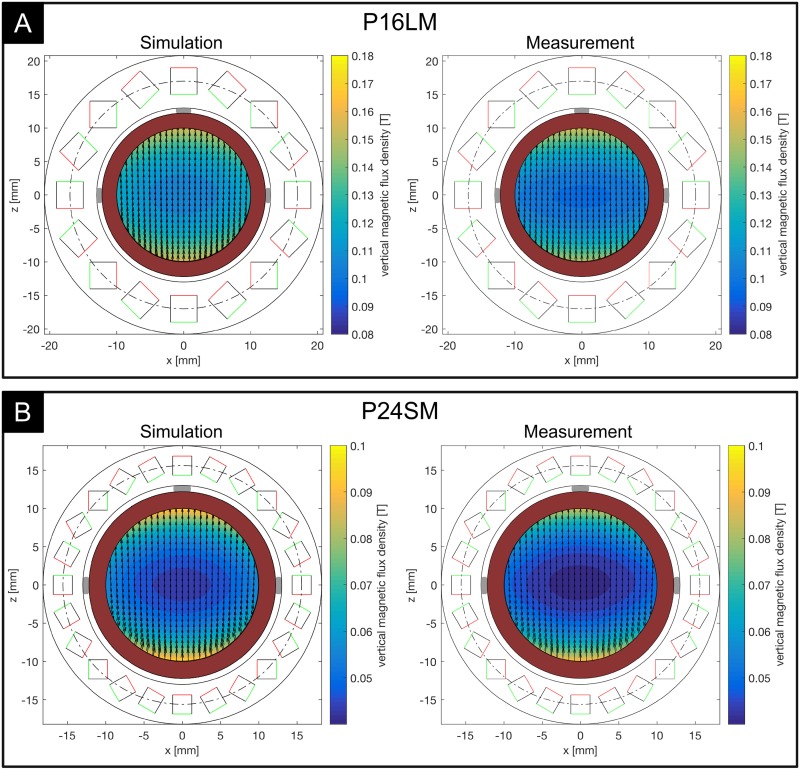
Results of magnetic field simulations and measurements in the center plane. Contours indicate the vertical magnetic flux density magnitude. Vectors indicate the in-plane magnetic field components. A: result for the 16-magnet prototype (P16LM). B: result for the 24-magnet prototype (P24SM). Gray blocks indicate the electrode positions. Red shaded region illustrates the blood vessel.

Fig 9 shows the magnetic field properties for P16LM (Fig 9A) and P24SM (Fig 9B). The contours indicate the center plane vertical magnetic flux density magnitude *B*_*z*_, and the vectors show the corresponding in-plane magnetic field components (*B*_*x*_,*B*_*z*_). The results confirm that a unidirectional magnetic field is created using the Halbach configurations with a predominantly vertical magnetic field with good agreement between simulation and measurement. The curvature of the magnetic field increased towards the outer regions of the array and close to the magnets. The vertical magnetic flux density magnitude showed considerable inhomogeneity along both axes, where the measured flux density dropped from the outer regions towards the center of the array. This effect was visible in both arrays and especially prominent for P24SM (Fig 9B). The maximum measured vertical magnetic flux density along the horizontal axis for P16LM was 107 mT, and it dropped to 100 mT in the center of the array. Along the vertical axis, the maximum was 155 mT and dropped to 100 mT. For P24SM, the maximum measured vertical magnetic flux density along the horizontal axis was 51 mT and dropped to 45 mT in the center. Vertically, the magnetic flux density dropped from 90 mT to 45 mT. In the simulations, the magnetic flux density dropped horizontally from 122 mT to 107 mT and vertically from 162 mT to 107 mT for P16LM. The decay in magnetic flux density for P24SM was from 59 mT to 45 mT horizontally and from 99 mT to 45 mT vertically, moving from the outer region towards the center. The drop in magnetic flux density from the outer regions towards the center was found to be mainly caused by the finite length of the arrays, and the effect was stronger in the second array as its magnets are only half the length of those used in P16LM [27]. These results present a deviation from the ideal Halbach array (of infinite extent) which produces a perfectly uniform magnetic field. In fact, it can be shown that a perfectly uniform magnetic field cannot be achieved using finite-length magnets [31]. Therefore, a trade-off exists between homogeneity of the magnetic field and size (i.e. length) of the magnet array. An important property of circular Halbach arrays in the present configuration is that the magnetic field is contained within the circular region inside the array. Outside the array, the magnetic field cancels out, such that is is exaclty zero for an ideal Halbach array. Using finite-length and finite-count magnets, the field outside the array does not vanish exactly, but drops off quickly at a short distance from the surface. The residual magnetic field for our configurations at a distance of 1 cm from the surface was on average 5.2% of the center magnetic flux density (<6 mT) for P16LM and 6.5% of the center magnetic flux density (<3 mT) for P24SM. Therefore, no critical interference with other implanted devices (e.g. pacemaker) due to residual magnetic fields is expected. No negative impact on the performance of the electronic circuit or the wireless communication could be observed.

### Electronic characteristics

**Fig 10:**
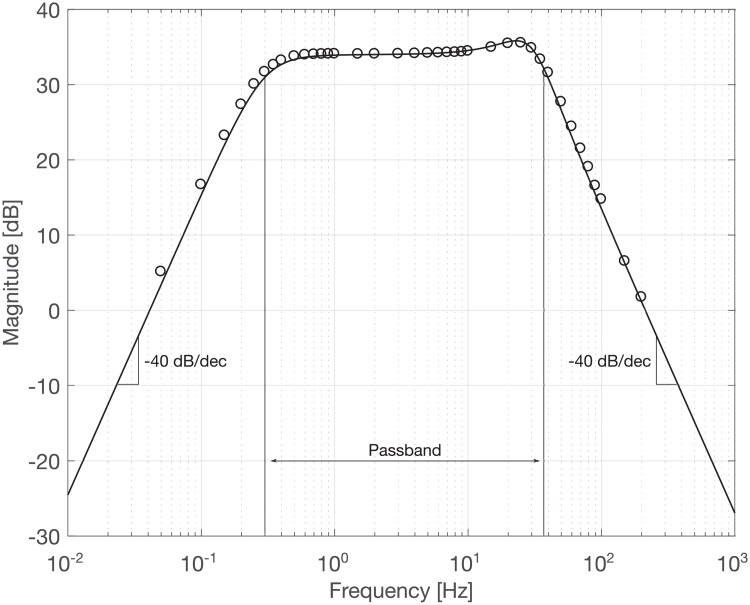
Results of transfer function measurement. Solid line: analytical solution. Circles: measurement values.

The results of the transfer function measurement are shown in Fig 10 which presents the frequency-dependent gain of the electronic circuit in a Bode plot. The circles mark the recorded test signal response for each test frequency. The solid line shows the analytical solution of the ideal transfer function, computed using nominal component values (resistors, capacitors, etc.). A good agreement between the measurement and the theoretical solution was found across the frequency range of interest. The highpass and lowpass filter stages produced the designed 40 dB/dec rolloff at cutoff frequencies of 0.3 Hz and 37 Hz, respectively. The signal showed amplification in the passband of 50 V/V (34 dB). It was observed that the signal attenuation in the low-frequency stopband was somewhat lower in the measurement when compared to the analytical solution. This may be attributed to residual measurement noise from insufficient averaging of the low-frequency signal response, leading to a slight overestimation of the signal amplitude. Overall, it could be confirmed that the implementation of the electronic circuit provided the designed frequency behavior.

### Flow benchmark measurements

**Fig 11:**
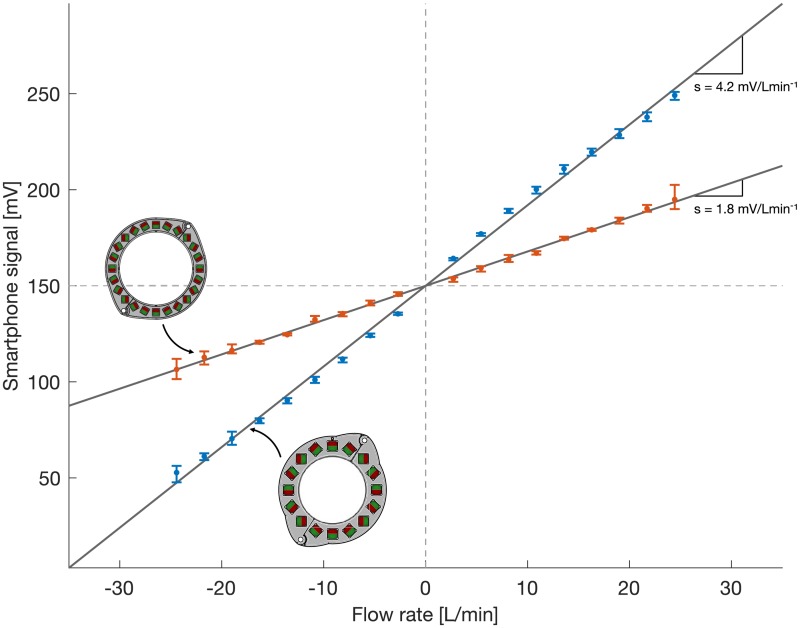
Results of flow benchmark measurements. Results for P16LM are shown in blue, those of P24SM are shown in red. The median signal amplitudes are plotted as dots, and the minimum and maximum measurement values are indicated with error bars to visualize repeatability. The lines depict the best linear fit through the measurement data.

**Fig 12:**
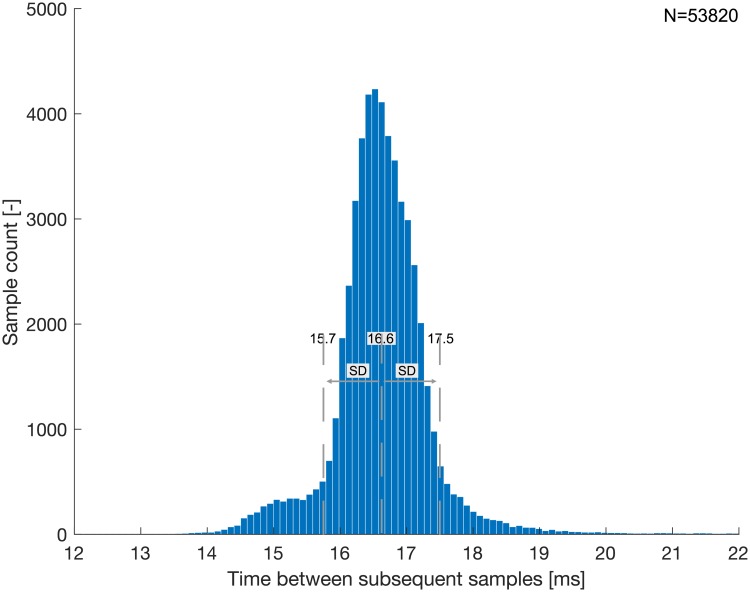
Analysis of effective sample rate. Histogram of the time between successive samples for the combination of all flow benchmark measurements. The dashed lines indicate the average time between samples and one standard deviation above and below the average.

The benchmark measurements in the flow setup depicted in Fig 8 were used to determine key characteristics of the sensor system which are summarized in Table 1. The results for both magnet rings are shown in Fig 11. The median signal amplitude of the measurements at each flow rate is shown as dots, and the maximum and minimum measurement values are shown as error bars. A linear fit through the measurement data of the form *y* = *Ax* was used to determine the sensitivity and the maximum nonlinearity of each sensor. The respective sensitivities were extracted from the slope of the linear fit (lines in Fig 11). The system using P16LM provided a sensitivity of 4.2 mV/Lmin^−1^. It was reduced to 1.8 mV/Lmin^−1^ for P24SM because the same amplification circuit with a gain of 50 V/V was used (at the reduced raw signal from the weaker magnetic field). The gain value of 50 V/V was chosen to yield a range of −35 L/min to 35 L/min using P16LM. For this study, the prototype using P24SM was equipped with the same amplifier which yielded an increased range at a reduced sensitivity. Here, we limited the measurement range of P24SM to match that of P16LM for easier comparison between the two. The resolution of the 10-bit ADC used was 0.29 mV at an input range of 300 mV. Together with the previously determined sensitivity, the resolution of the flow sensor could be computed as 0.070 L/min for P16LM and 0.163 L/min for P24SM, respectively. The maximum nonlinearity was determined from the maximum median deviation of the measurement values from the linear fit and was computed as

$$\delta_{y}=\frac{\text{max}|y(x)-Ax|}{A(x_{\text{max}}-x_{\text{min}})},$$ (12)

where *y*(*x*) is the median measurement value at the flow rate *x* and *Ax* presents the ideal value at *x* according to the linear fit. The maximum nonlinearity for the system using P16LM was 1.8% of full scale. That of the P24SM system was 1.9% of full scale (±35 L/min). The repeatability (i.e. the spread) of the smartphone recordings was evaluated by computing length of the error bars (maximum and minimum measurement value) for each set of measurements and by dividing by the corresponding median value. The median spread over the range of flow rates using P16LM was 2.0% and that using P24SM was 1.7%, respectively. The noise level was at 0.83 mV_rms_ for both prototypes. Fig 12 shows the time between successive wirelessly transmitted samples for all flow benchmark measurements combined. The average time between successive samples was 16.6 ± 0.9 ms, resulting in an effective sample rate of 60.2 ± 3.3 Hz.

**Table 1:** Key characteristics of the two sensor prototypes P16LM and P24SM as determined from the flow benchmark measurements.

	P16LM	P24SM
range	−35 L/min …35 L/min	−167 L/min … 167 L/min
sensitivity	4.2 mV/Lmin^−1^	1.8 mV/Lmin^−1^
resolution	0.070 L/min	0.163 L/min
nonlinearity (max)	1.8%	1.9%
spread (med)	2.0%	1.7%
noise	0.83 mV_rms_	0.83 mV_rms_
sample rate (avg)	60.2 Hz	60.2 Hz
power consumption	1.3 mW	1.3 mW

**Fig 13:**
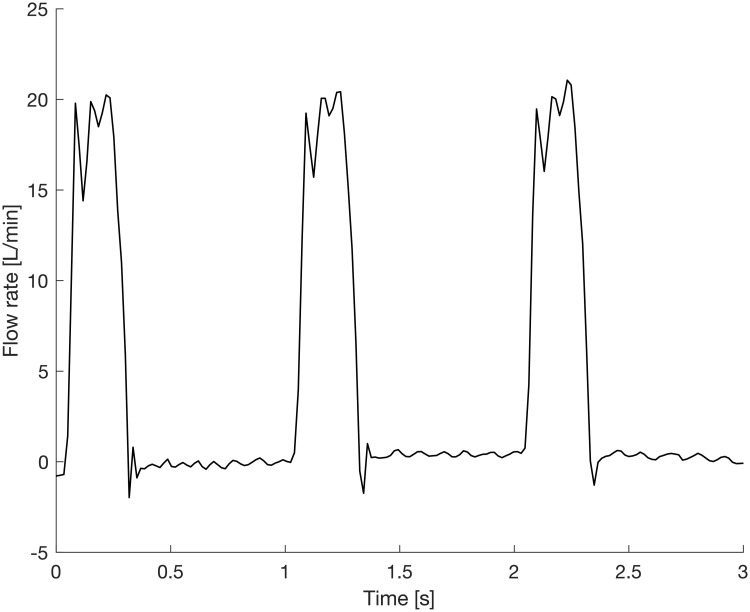
End-to-end measurement in a physiological flow loop. A flow loop produces physiological flow (cardiac output: 5 L/min, systolic to diastolic pressure: 120/80 mmHg) and the instantaneous flow rate through the ascending aorta is recorded with the sensor system.

Finally, Fig 13 shows a measurement of the flow in the ascending aorta, produced by an in-vitro flow loop [32] set to physiological conditions (heart rate: 60 BPM, cardiac output: 5 L/min, systolic to diastolic pressure: 120/80 mmHg) and using P16LM. The highly unsteady nature of the temporal flow rate profile in the aorta and ambient noise gave rise to some signal fluctuations, but the most prominent features of the aortic flow rate profile could be resolved, including peak systolic flow, the closing volume related to the valve kinematics, and any potential diastolic leakage.

Such flow recordings can be used to create a statistical baseline model of the patterns of blood flow downstream of the valve under physiological conditions. When acquired in-vivo, it includes the physiological cycle-to-cylcle variability from slightly different loading conditions between heartbeats. A pathology is detected when blood flow recordings persistently and progressively deviate from the baseline by more than the physiological variability (which is included in the model) [28]. A direct link between the level of model deviation and predefined valvular pathologies does not yet exist, but may be established during clinical trials.

## Discussion

Magnetic fields of considerable strength could be created using permanent magnet rings. This in turn created raw signals of relatively high amplitude which facilitated the signal conditioning and allowed for low electronic gain. The strong magnetic field created even with the small magnet ring suggests that further miniaturization is possible while maintaining an adequate signal-to-noise ratio. However, the properties of the magnetic fields were not truly homogeneous. While the effect of field inhomogeneity can be mostly eliminated through calibration, the ideal magnetic flow meter assumes a perfectly homogeneous magnetic field throughout the measurement volume. Under these conditions the flow meter reading is independent of the velocity distribution within the measurement volume as long as the velocity profile has rotational symmetry [23]. The assumption of a homogeneous magnetic field does not hold for our prototypes, and a certain dependency of the flow measurement on the underlying velocity profile has to be expected. Still, this effect produces errors that are much smaller than one might expect from the inhomogeneity of the magnetic field, because the infinitesimal errors in the measurement volume partially cancel out [27]. The flow conditions downstream of the AV (turbulent, vortical, unsteady) are challenging for any type of flow meter and accuracies found in well-controlled industrial applications cannot be matched in the human body. Factors, such as flow pulsatility, turbulent fluctuations and vortical velocity components which propagate from the ventricle into the ascending aorta lead to very complex flow profiles that undergo various morphological changes during the cardiac cycle. This introduces some inaccuracies in the flow meter readings (which often assume a parabolic velocity profile). While magnetic flow meters are among the technologies which are least affected by the velocity profile, the measurements in the physiological flow loop show that additional flow-induced fluctuations are introduced. Absolute accuracy, however, is of lesser concern for AV monitoring as these systems aim at detecting relative changes in flow behavior, rather than analyzing the absolute flow rate of a single recording. Repeatability is therefore a more important metric for monitoring systems, and the results of the benchmark measurements suggest that reliable measurements can be obtained using the implant. The electronic circuit delivered its designed functionality and the signal could be successfully processed for quantization and wireless data transmission. The power consumption was within the power budget such that batteryless operation could be sustained as long as the external RF-field was supplied. We believe that the temporal resolution of the sensor system presents the most important limiter of device performance. Still, while a higher sample rate certainly would be desirable and appears technically achievable, the effective sample rate of 60.2 Hz proved to be sufficient to capture all relevant features of the flow in the ascending aorta. The sample rate was mostly limited by the speed at which the NFC chip could process the smartphone requests and compile the response message. A more advanced NFC chip could therefore yield improvements with respect to the temporal resolution. The full measurement system showed promising characteristics with respect to resolution, nonlinearity, repeatability and sample rate to enable blood flow measurements at a confidence level sufficient to detect anomalies in the flow behavior downstream of the AV.

## Limitations and future work

The functional characterization of the sensor prototypes was assessed in a laboratory environment exclusively. The results of these test were promising and indicate that physiological flow signals in a blood vessel may be recorded with the proposed technology. Still, further tests in a more clinical setting (e.g. in animal trials) are necessary to validate the system’s performance in a more realistic application environment. Biocompatibility was considered through a choice of a biocompatible frame material (TiAl6V4), but to a large degree biocompatibility is determined by the device’s encapsulation. Such encapsulation was not implemented in the current prototypes and needs to be added before long-term clinical animal trials can be performed. This may be achieved using biocompatible metals, ceramics or polymers. The most promising approach appears to be silicone coating as it ensures biocompatibility, has a smooth texture that accomodates complex shapes, and provides a hermetic seal for the sensitive electronics. Similarly, the long-term measurement stability of the system which may be affected by tissue ingrowth was not investigated. The prototypes discussed in this article were designed for a vessel diameter of approximately 26 mm. More size options need to be developed for clinical applications to accommodate different vessel sizes and to ensure a good implant-vessel match.

## Conclusion

A sensor system was developed to monitor prosthetic heart valve function by means of wireless and batteryless blood flow measurement. A modification of the classical electromagnetic flow meter using permanent magnets in a Halbach ring configuration allowed reducing the power requirements of the device to less than 2 mW. Wireless and batteryless operation could be sustained while an NFC-enabled smartphone was in the vicinity of the implant and transmitting power through inductive coupling. An effective sample rate of 60.2 Hz. could be achieved which is sufficient to capture the most relevant flow features. The magnet ring could be miniaturized to an implantable form factor at the cost of reduced magnetic field homogeneity. The benchmark measurements suggest that the device can deliver reliable flow recordings, but some compromises regarding sensor performance need to be accepted when compared to industrial flow meters (which are much larger in size and typically operate in a well-controlled environment). Overall, the results of our investigations suggest that the proposed measurement system can meet specifications suitable for remote prosthetic heart valve monitoring and may present one approach of how sensory implants, coupled with consumer smartphones, can facilitate the management of cardiovascular diseases.

## Supporting information

S1 Dataset P16LM magnetic field measurement. (MAT)

S2 Dataset P16LM magnetic field simulation. (MAT)

S3 Dataset P24SM magnetic field measurement. (MAT)

S4 Dataset P24SM magnetic field simulation. (MAT)

S5 Dataset Filter transfer function measurement. (MAT)

S6 Dataset P16LM sinusoidal flow benchmark measurement. (MAT)

S7 Dataset P24SM sinusoidal flow benchmark measurement. (MAT)

S8 Dataset P16LM physiological flow measurement. (MAT)
